# Recurrence of Early Stage Colon Cancer Predicted by Expression Pattern of Circulating microRNAs

**DOI:** 10.1371/journal.pone.0084686

**Published:** 2014-01-06

**Authors:** Narayan Shivapurkar, Louis M. Weiner, John L. Marshall, Subha Madhavan, Anne Deslattes Mays, Hartmut Juhl, Anton Wellstein

**Affiliations:** 1 Lombardi Cancer Center, Georgetown University, Washington, District of Columbia, United States of America; 2 Indivumed GmbH, Hamburg, Germany; Baylor University Medical Center, United States of America

## Abstract

Systemic treatment of patients with early-stage cancers attempts to eradicate occult metastatic disease to prevent recurrence and increased morbidity. However, prediction of recurrence from an analysis of the primary tumor is limited because disseminated cancer cells only represent a small subset of the primary lesion. Here we analyze the expression of circulating microRNAs (miRs) in serum obtained pre-surgically from patients with early stage colorectal cancers. Groups of five patients with and without disease recurrence were used to identify an informative panel of circulating miRs using quantitative PCR of genome-wide miR expression as well as a set of published candidate miRs. A panel of six informative miRs (miR-15a, mir-103, miR-148a, miR-320a, miR-451, miR-596) was derived from this analysis and evaluated in a separate validation set of thirty patients. Hierarchical clustering of the expression levels of these six circulating miRs and Kaplan-Meier analysis showed that the risk of disease recurrence of early stage colon cancer can be predicted by this panel of miRs that are measurable in the circulation at the time of diagnosis (P = 0.0026; Hazard Ratio 5.4; 95% CI of 1.9 to 15).

## Introduction

Colorectal cancer (CRC) is the third leading cause of cancer mortality that affects men and women equally. Worldwide it accounts for approximately one million new cancers and one-half million deaths representing 10 percent of cancer deaths [1]. Outcomes for patients with early-stage CRC are heterogeneous, with disease-specific 5-year survival rates for patients with stage II of 72–88% and 40–71% for stage III [2]. Most patients with stage II disease are cured by surgery alone whilst additional chemotherapy can provide survival benefits to patients with later stage disease. Still, approximately 1 in 4 patients with early stage disease will suffer from recurrence and biomarkers that identify patients at high risk at the time of initial diagnosis and surgery would allow to select those patients for closer monitoring and possibly systemic treatments ([3], [4], [5]; reviewed recently in [6]).

microRNAs (miRNAs or miRs) are small, non-coding RNAs that play a significant role in controlling the activities of cellular pathways both in physiology and pathology (see e.g. [7]). The distinct function of miRs in different cancers has become more obvious over the past years [8], [9], and many studies show that miR signatures can be used to distinguish different cancers [10], [11], [12], [13], prognoses [14], [15], reveal potential targets [16], altered signaling pathways [17], or malignant progression from in situ carcinoma to invasive disease [18]. Most surprisingly, a comparison of miR and mRNA profiles of primary and metastatic cancer lesions showed that miRs provide a more reliable and distinctive signature than mRNAs and found that miR signatures were superior to mRNAs in identifying the organ source of metastases of unknown origin [19], [20]. A previous study [21] using expression profiling of 315 miRs in tissues from stage II colon cancers showed differences in the patterns for recurrent disease. The expression levels of specific miRs in the tissues correlated with the probability of recurrence-free survival by multivariate analysis. These results suggest that perturbed expression of miRs in colon cancer may have a prognostic potential.

Beyond these analyses of normal and diseased tissues, more recent reports have shown that miR species can be detected in the circulation [22] and even suggested that analysis of serum samples for defined miRs could be used to identify patients with cancers [23], [24], [25], [26]. Studies of the blood-borne miRome also showed applicability to diseases other than cancer ([27]; reviewed in [28], [29]). miRs in the circulation appear to be remarkably stable [30], [31] and it was suggested that the stability is attributable to miRs being included in lipoprotein vesicles, known as exosomes [32]. Here, we studied miRs in blood samples obtained pre-surgically from patients with early stage colon cancer that remained recurrence-free or had disease recurrence. We hypothesized that altered patterns of circulating miRs might indicate an increased risk of disease recurrence due to occult metastatic seeds at the time of initial diagnosis. We report that a set of six miRs may be useful in predicting the risk disease recurrence for early stage colon cancers.

## Results

### Circulating microRNA Expression Comparison Using a Candidate Gene Approach

**Figure 1:**
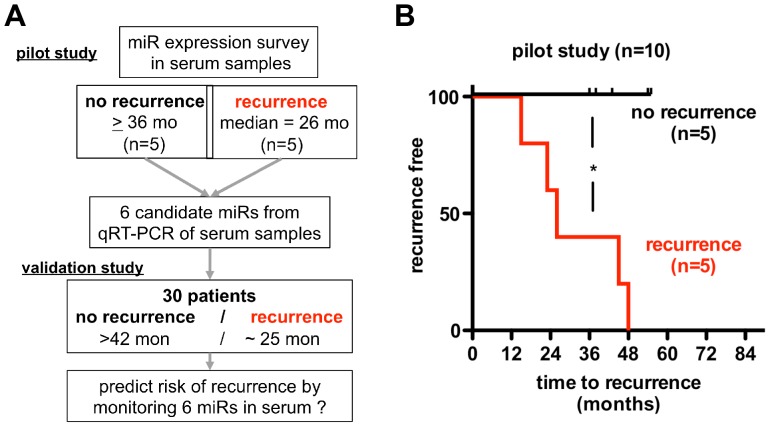
Study design (A) and time-to-disease recurrence in early stage colon cancer patients in the pilot study (B). **A**, From a pilot study with 10 patients candidate miRs predictive of disease recurrence were identified and tested for their prediction of disease recurrence in a validation study. **B**, Kaplan-Meier plot of disease recurrence in patients in the pilot study. Patients with disease recurrence (n = 5) vs no recurrence (n = 5): Chi square 5.47, p = 0.0193; median time-to-recurrence = 26 months. The Gehan-Breslow-Wilcoxon algorithms were used.

**Figure 2:**
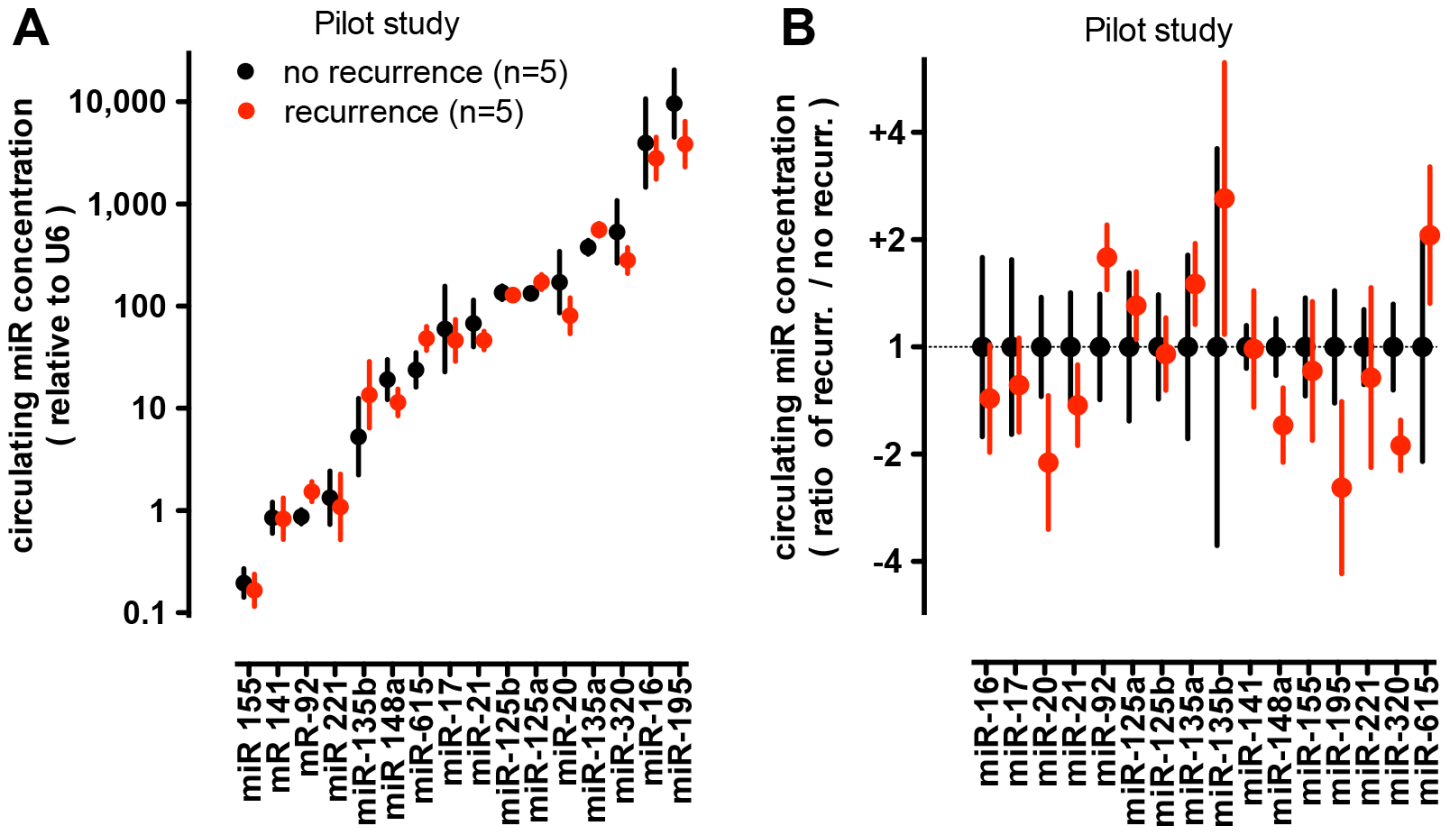
miR expression levels in serum samples from patients with or without recurrence of early stage colon cancer (Pilot Study). Candidate miR approach. Circulating levels of 16 miRs indicated on the x-axis that had been published as differentially expressed between colon cancer and non–malignant colon tissues were analyzed [21], [34], [35]. Pre-surgery serum samples were from patients in the pilot study. Patients had been followed for disease recurrence and the respective data are in Fig. 1B. **A**, Concentration of circulating miRs (relative to U6). Note the log-scale that covers a range of 100,000-fold. **B**, ratio of expression between patient groups. Alhough miR-20, miR-195 and miR-320 showed a ≥2-fold downregulation, and miR-135b and miR-615 a ≥2-fold upregulation in serum from patients with disease recurrence, neither of the comparisons reached statistical significance by ANOVA (p>0.05).

To measure miRs in the circulation, we established quantitative RT-PCR [33] as a detection method with a dynamic range up to 10^6^-fold for miRs in serum samples [18]. As an initial approach, we picked a panel of sixteen miRs that had been shown to be differentially expressed between colon cancer and normal colon tissues [21], [34], [35]. We initially ran a pilot study and analyzed a set of serum samples from early stage colon cancer patients that remained disease-free (n = 5) or had disease recurrence (n = 5) within an average of 26 months (<0.05 vs. no recurrence; Fig. 1A,B). Fig. 2A shows the approximately 100,000-fold concentration range of the sixteen circulating miRs analyzed in the pilot study. We observed the expected trends of the respective up- or down-regulation in some of the selected miRs (i.e. miR-20, miR-135b, miR-195, miR-320, miR-615). However, the differences were not statistically significant (Fig. 2B). It is noteworthy that miR-320 was one of two miRs that showed a downregulation in tissues that was correlated with poor recurrence-free survival [21]. Here, miR-320 in the circulation was approximately two-fold lower in patients with disease recurrence, though that downregulation was not statistically significant (Fig. 2B). The expression of miR-498, the other miR from the study in Ref. [21], was detected only at very low levels in the circulation and thus not suitable for the analysis.

### Circulating microRNA Expression Comparison using an Unbiased, Genome-wide Analysis

**Figure 3:**
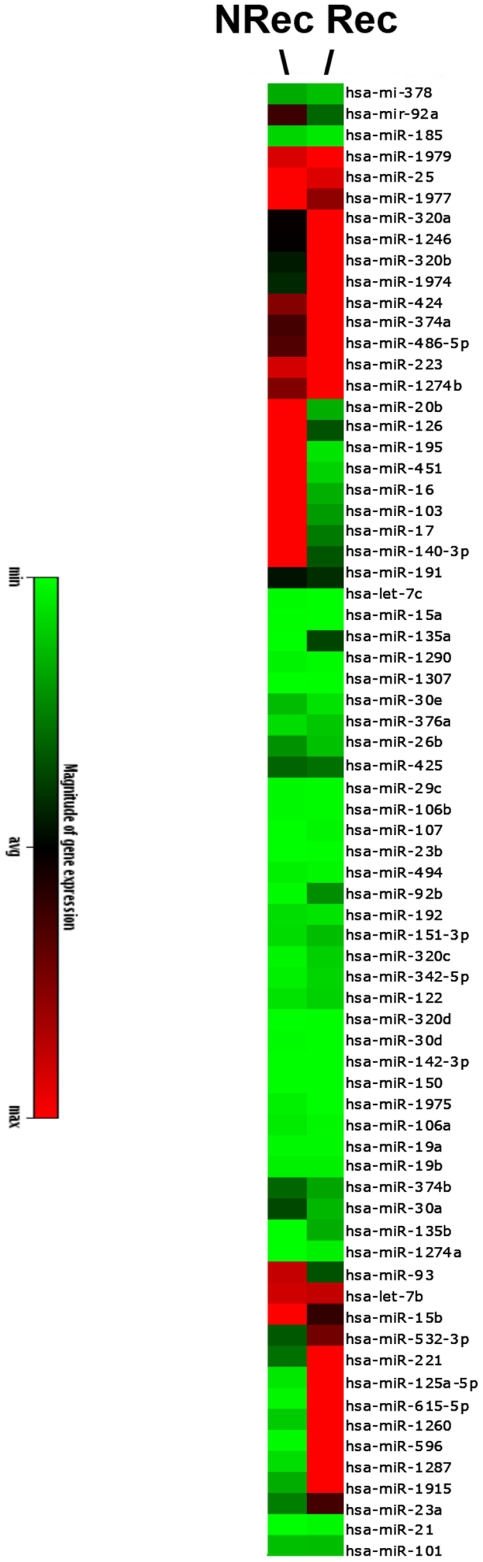
miRNA expression levels in serum samples from two patients with (Rec) or without (Nrec) 5-year recurrence of early stage colon cancer. Heat map of genome-wide microRNA expression analysis using quantitative RT-PCR. Two patients were selected from the pilot study (see Figs. 1 and 2). The miRNA expression analysis was carried out for 760 miRNAs using qPCR-based expression analysis. Data filtration and analysis were conducted and the 70 miRs that show the biggest differential expression are represented in the heat map.

From the above pilot study we selected two patient samples from the recurrence-free (NRec) and recurrence (Rec) group for an unbiased genome-wide miR analysis. We picked patients from the above pilot study that had shown the most differential expression of candidate miRs. We reasoned that a comparison within such a sample pair might offer a greater chance of picking up recurrence-specific miRs. We analyzed this pair of patient samples using SYBR Green qPCR based genome-wide miR expression arrays for 760 miRs set up in our laboratory, using commercially available reagents (SBI, Mountain view CA). The respective result for the top 70 miRs is shown in Fig. 3 as a heatmap. For an independent validation study we selected from this panel six miRs that met two criteria: (1) sufficient levels of expression in serum samples, i.e. RT-PCR Ct values ≤32 and (2) differential expression (>3-fold up- or down-regulation) of a given miR comparing Rec and NRec samples.

### Validation of Circulating microRNA Expression Patterns as a Predictor of Disease Recurrence

To evaluate whether the circulating miRs identified in the above pilot study can predict disease recurrence in low-risk patients, we used a separate, independent group of thirty patients with early stage colon cancer with known outcomes. Fifteen of these patients had disease recurrence within an average of 25 months whereas the other fifteen remained recurrence-free. The clinical characteristics of patients in this validation set at the time of their initial diagnosis is provided in Table 1. At the time of diagnosis the two groups of patients without and with disease recurrence showed no significant differences with respect to age, gender, tumor size, tumor stage, location of their primary lesion or histopathology (Table 1). All patients had node-negative disease and more than twelve lymph nodes examined. Only one patient (in the non-recurrence group) was a T4, all others were T1 to T3 tumors and thus with a low known risk of recurrence.

**Table 1:** Patient characteristics in the validation study.

parameter	recurrence (n = 15)	no recurrence (n = 15)	P-value (across groups)
age (years)	69.5±2.4	62.5±3.1	P>0.05
gender (female/male)	3/12	9/6	P>0.05
tumor size (cm)	3.8±0.4	3.9±0.3 cm	P>0.05
stage: T1/T2/T3/T4	2/2/11/0	1/7/6/1	P>0.05
lymph nodes (positive out of median number examined [range ])	0/16 [12 to 54]	0/24 [13 to 58]	P>0.05
location (ascending & transverse/sigmoid/rectal )	5/5/5	4/8/3	P>0.05
histopathology (moderately/poorly differentiated)	12/3	14/1	P>0.05
leukocytes (cells/nl)	7.8±1.9	6.0±0.3	P>0.05
CEA (ng/ml) [median; 5%–95% C.I.]	2.8 [1.0–63.8]	1.6 [0.7–4.8]	P>0.05

**Figure 4:**
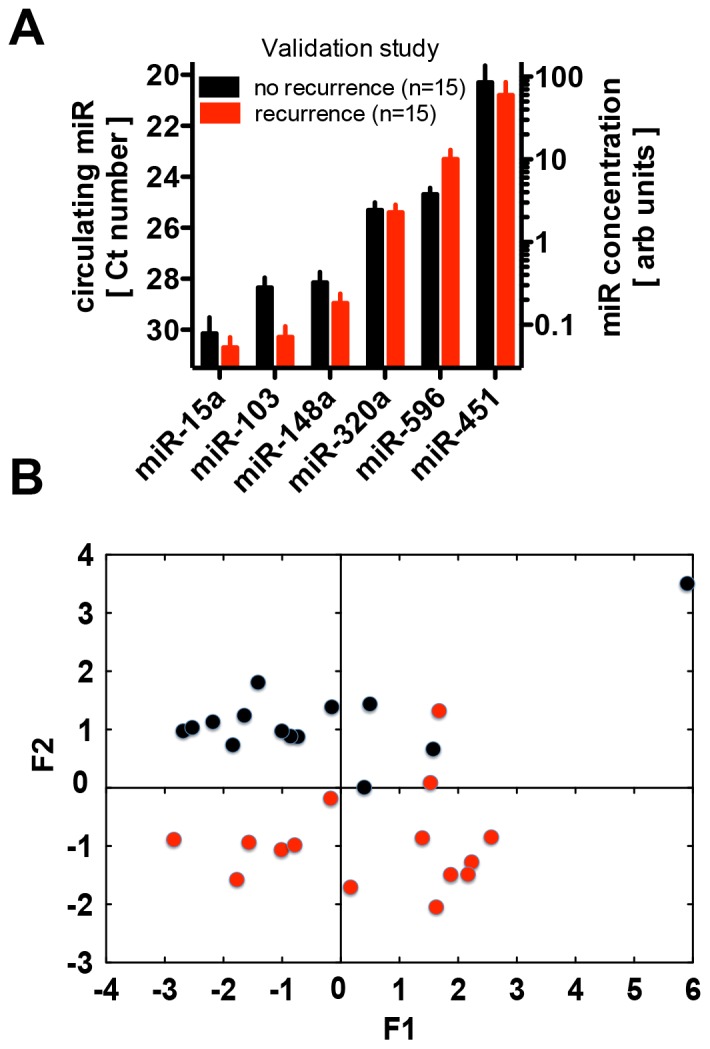
miR expression levels in serum samples from patients with or without recurrence of early stage colon cancer. Validation study for six miRs identified in the Pilot study. Patient characteristics are provided in Table 1. Six miRs were derived from the pilot study. **A**, Expression levels based on the cycle threshold (Ct) values of the qRT-PCR (left axis) and the miR concentration calculated (right axis). **B**, Principal Component Analysis of the data with the two groups shown in black and red symbols respectively.

Expression of the six miRs derived identified in the pilot study was measured by qRT-PCR and the resulting data are shown as raw Ct values as well as a concentration range in Fig. 4A (left and right axes respectively). The concentration of the six selected miRs in the circulation covers a range of approximately 1,000 fold, and thus a 100-fold narrower range than the miRs monitored in the pilot study (compare Figs. 4A and 2A). A correlation analysis of the data showed a strong correlation of the expression levels of miR-15a, miR-148a, miR-320a and miR-451 (r = 0.746 to 0.897; p<0.0001) whereas levels of miR-103 and miR-596 were not correlated with the levels of the other miRs. A Principal Component Analysis (PCA) of the data set showed a distinct grouping of patients with and without recurrence (Fig. 4B) suggesting that the panel of miRs may distinguish the different recurrence risks of patients.

**Figure 5:**
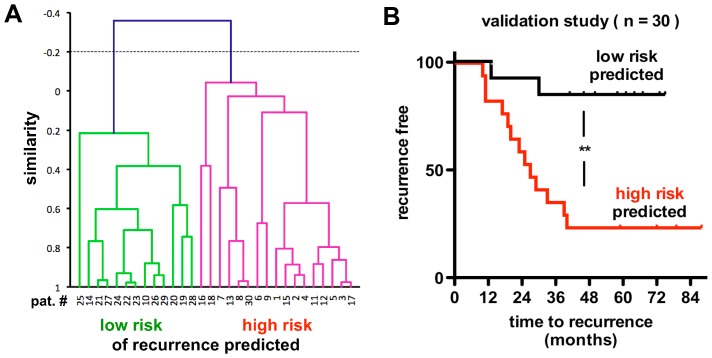
Disease recurrence in patients relative to their risk predicted from the serum levels of miRs. **A,** Hierarchical clustering of patients from the validation study into low and high risk groups based on serum miR levels. The analysis resulted in a separation into two groups. **B**, Kaplan-Meier plot of patients predicted to have high and low risk of disease recurrence. The comparison resulted in P = 0.0026, Hazard Ratio 5.4 (1.9 to 15.0 95% CI). In panel A patients with disease recurrence are #1 to #15 and without recurrence: #16 to #30.

A blinded hierarchical clustering analysis [18] (Fig. 5A) showed a significant subsetting into two distinct groups. Thirteen of fifteen patients with disease recurrence and eleven of fifteen patients that were recurrence-free were correctly classified by the miRs in the blood samples collected before their initial surgery (p = 0.0025; odds ratio = 17.9; rel. risk = 5.5, 95%CI = 1.5 to 20.7). A Kaplan-Meier analysis of the times to disease recurrence in the patients assigned to high or low risk by the hierarchival clustering showed a significantly different outcome between the two groups (P = 0.0026, Fig. 5B). Thus, the six miRs selected here can be used to predict the risk of disease recurrence of early stage, low-risk colon cancer by the analysis of a blood sample collected at the time of the initial diagnosis.

## Discussion

The current study evaluates the possible use of circulating microRNAs (miRs) to identify colon cancer patients with a high risk of disease recurrence inspite of a diagnosis of early stage, low-risk disease indicated by established tumor staging. This could help identify those individuals that would benefit from closer surveillance and/or additional systemic therapy and would avoid over treatment of patients that will not benefit due to their low risk (reviewed recently in [6]). The analysis can be done with a small amount (<1 ml) of serum.

The origin of circulating miRs has been a subject of debate. Tumor-associated miRs detected in the circulation could obviously result from tumor cell death as well as from active release by tumor cells. Furthermore, miRs packaged in protective carriers such as exosomes can be delivered to recipient cells where they exert gene silencing through the same mechanism as cellular miRs [36] or can be shed into the circulation. Furthermore, secreted miRs have been hypothesized to be involved in mediating cell-cell communication. Also select sets of miRs are released by cancer cells and the composition of these sets of miRs has been related to malignancy [37]. Although cancer-associated circulating miRs are can be derived from cancer cells themselves, immune or other stromal cells in the tumor microenvironment as well as other organs that respond to signals from the tumor are possible sources [38]. Thus, immune cells may secrete cancer-associated miRs, thereby promoting or inhibiting proliferation, invasion and apoptosis [38], [39] and dysregulation of these miRs may be an important link between immunity and cancer. Although, some studies found parallel trends between circulating miRs and tissue miRs (e.g. [18], [24]) others suggest that not all the released miRs reflect the miR abundance in tumor cells (e.g. [37], [40]). In general, miRs detected in the circulation are provided by different organs and physiologic regulation or pathologic changes will alter the expression patterns of circulating miRs (for recent examples and reviews see Refs. [29], [41], [42], [43]).

When analyzing the miRs in the current study one-by-one rather than in a multivariate analysis, two miRs were found differentially expressed in the circulation, i.e. miR-103 (downregulated) and miR-596 (upregulated). miR-103 was significantly down-regulated (p = 0.038) in blood samples from patients with disease recurrence compared to recurrence-free patients (Fig. 4A). In intestinal crypt cells miR-103 is part of the G1/S transition regulatory network during IGF-1 stimulated proliferation and this points to a significant role in cell growth and survival [44]. In contrast, miR-596 was significantly up-regulated (p = 0.0012) in blood samples from patients with disease recurrence. Expression of miR-596 was correlated with poor survival of patients with ependymoma [45] but was inactivated in HCC [46] and downregulated in pancreatic adenocarcinoma [47]. Thus, there is no clear evidence yet to link tissue expression of this miR to a distinct functionality that would connect it to disease progression. miRs with only small overall changes between the patient groups were miR-148a that is part of a group of negative regulators of pro-inflammatory genes in myeloid cells [48], yet is decreased in pancreatic cancer tissues [18], expressed at high levels in liver tissues [49] and associated with liver injury [50]. miR-320a was reported earlier as a promising indicator of malignant progression in colon cancer [25] though we did not see a clear indication of the impact of miR-320 changes on their own in this study. miR-15a can cause apotosis [51] and has been reported to participate in the control of tumor-stromal crosstalk in prostate cancer [52] as well as mediate proangiogenic pathways in ischemia-associated pathology [53]. Interestingly, miR-451 was characterized as a circulating indicator of the presence of breast cancer [54] and was shown to be selectively released from transformed mammary epithelia and to contribute to altered sensitivity to chemotherapeutic or endocrine targeted drugs in breast cancer (reviewed in [55]). It is tempting to speculate that the circulating miRs identified here as indicators of the risk of disease recurrence may contribute to the process by impacting tumor cell survival, tumor stromal interactions, angiogenesis or inflammatory cell responses to the occult metastases.

### In Conclusion

The risk of recurrence of early stage colon cancer may be predicted by monitoring miRs in patient blood samples collected at the time of the initial diagnosis.

## Materials and Methods

### miR Detection and Quantitation in Blood Samples

The collection and use of biospecimen was approved by the Institutional Review Board (IRB) of Georgetown University under Protocol #2007-345 most recently on 10/24/2012. All patients sign a consent form permitting the use of donated tissue and body fluid samples. The consent forms and their content was reviewed and approved by the IRB. Serum samples (<1 ml) were processed after removal of personal identifiers. miR isolation was described previously [18]. In brief, serum samples were mixed at a ratio of 1∶10 with Qiazol lysis reagent and vortexed. The lysate was extracted with CHCl3 and the aqueous phase was further processed for total RNA using the miRNeasy Mini kit (Qiagen, Valencia, CA) and enriched for miR using the RT2 qPCR-Grade miR Isolation Kit, MA-01 (SABiosciences).

### Data Analysis

PCA and hierarchical clustering were performed based on the mean centered and scaled miR expression levels. The clustering methods used XLSTAT (Addinsoft Inc.) within Excel (Microsoft Inc.) on OSX 10.7.5. These methods allow for the calculation of significance between the hierarchical clusters and derive p-values using Fisher's exact test. Prism 5.0 (Graphpad) software was used for other tests and display of the data.
