# Correction: Recurrence of Early Stage Colon Cancer Predicted by Expression Pattern of Circulating microRNAs

**DOI:** 10.1371/annotation/86f9cc5c-58b7-4c07-8742-c3036c13e73a

**Published:** 2014-01-16

**Authors:** Narayan Shivapurkar, Louis M. Weiner, John L. Marshall, Subha Madhavan, Anne Deslattes Mays, Hartmut Juhl, Anton Wellstein

Information regarding the first and last authors is incorrectly omitted from the Competing interests statement. The Competing interests statement should read: 

"One of the authors (Dr. Juhl) is affiliated with Indivumed, GmbH. A provisional patent application has been filed by Georgetown University related to the technology described in this paper. Drs. Wellstein and Shivapurkar are named as inventors on this provisional patent application.This does not alter the authors’ adherence to all the PLOS ONE policies on sharing data and materials and does not provide a conflict of interest."

